# Low-Molecular-Weight Salmon Collagen Peptides Promote Extracellular Matrix Gene (ECM) Expression in BJ Fibroblasts

**DOI:** 10.3390/ijms27188090

**Published:** 2026-09-11

**Authors:** Soottawat Benjakul, Krisana Nilsuwan, Umesh Patil, Thaiyawat Haewphet, Jirakrit Saetang

**Affiliations:** International Center of Excellence in Seafood Science and Innovation, Faculty of Agro-Industry, Prince of Songkla University, Hat Yai 90110, Songkhla, Thailand; soottawat.b@psu.ac.th (S.B.); krisana.n@psu.ac.th (K.N.); umesh.p@psu.ac.th (U.P.); thaiyawat.h@psu.ac.th (T.H.)

**Keywords:** fish skin, cosmeceuticals, extracellular matrix, collagen peptides, bioeconomy, fibroblast

## Abstract

Salmon skin, a high-volume by-product of seafood processing, offers a circular-bioeconomy route to sustainable, value-added ingredients. This study aimed to generate low-molecular-weight collagen peptides (LMWCPs) from salmon skin using a stepwise enzymatic process and to evaluate their safety and pro-extracellular-matrix (ECM) activity in human BJ fibroblasts. LMWCPs were produced by sequential hydrolysis (alcalase/papain, then collagenase) and characterized as low-molecular-weight peptide preparations with a mean dispersed particle diameter of approximately 117 nm. LMWCPs display negatively charged peptide dispersions with a mass centered around ~1 kDa. Cytocompatibility (MTT) showed no toxicity up to 1.5 mg/mL over 48 h. Gene expression by reverse transcription–quantitative polymerase chain reaction (RT-qPCR) revealed a robust, dose-dependent ECM response: collagen type I alpha 1 chain (COL1A1) increased by approximately 7-fold, with additional rises of 5–6-fold in versican (VCAN) levels and modest increases in elastin (ELN) and transforming growth factor-β (TGF-β). These findings provide an exploratory process-to-phenotype link between the two-step hydrolysis process, physicochemical characteristics of the resulting LMWCPs, and changes in ECM-related gene expression in BJ fibroblasts. Overall, this study demonstrates that salmon skin LMWCPs were cytocompatible within the tested concentration range and modulated several ECM-related genes, providing a preliminary basis for future protein-level, functional, and translational evaluation.

## 1. Introduction

Skin biomechanics arise from cell–cytokine–growth factor crosstalk within an extracellular matrix (ECM) scaffold [1]. Fibril-forming type I collagen is the predominant type, accounting for approximately 90% of skin collagen, and underpins the structural organization, tensile strength, and mechanical integrity [2]. In the dermis, collagen fibrils support the epidermis, while elastin/microfibrils confer elasticity and resilience [1]. Dermal fibroblasts, stimulated by transforming growth factor-β (TGF-β), produce type I collagen, elastin, and proteoglycans, thereby regulating ECM expression, deposition, and turnover [3]. Aging-related ECM deterioration reflects both intrinsic drivers (oxidative stress, telomere/hormonal changes) and extrinsic insults, including UV/blue light-induced ROS upregulation, pollution/ozone-mediated NF-κB/IL-8 and MMP-9 increase, smoking dampening TGF-β1 signaling, or collagen stiffening via dietary glycation [4]. These factors can lead to collagen fibrils fragmenting and declining, reflecting reduced synthesis and enhanced degradation of type I collagen encoded by the COL1A1 gene [5,6]. Elastin (ELN) also undergoes increased breakdown, and versican (VCAN) forms loose, hydrated matrices that modulate adhesion, survival, proliferation, migration, and ECM assembly. These scenarios collectively contribute to architectural and aging-related changes [2,7]. Most previous studies focused on collagen or elastin endpoints alone. Versican and the upstream TGF-β axis, which govern dermal matrix assembly, hydration, and remodeling, remain under-profiled in collagen peptide research. Although such mechanisms are increasingly well understood, there remains interest in identifying marine-derived collagen hydrolysates capable of modulating ECM-related responses in dermal fibroblasts.

Bioactive peptides have become increasingly relevant as cosmetic active ingredients because their biological activities are strongly influenced by amino-acid sequence, molecular size, charge, and other physicochemical properties. Cosmetic peptides are commonly classified according to their mechanisms of action as signal peptides, carrier peptides, neurotransmitter-inhibitory peptides, and enzyme-inhibitory peptides [8,9]. Signal peptides are primarily associated with modulation of fibroblast and extracellular matrix-related pathways, whereas carrier peptides facilitate the delivery of trace elements, neurotransmitter-inhibitory peptides modulate neuromuscular signaling, and enzyme-inhibitory peptides can interfere with enzymes involved in matrix degradation [8,9]. The effectiveness of peptides in topical formulations, however, is also influenced by physicochemical factors such as molecular size, solubility, stability, and skin permeability, and improving peptide delivery across the stratum corneum remains an important area of cosmetic research [8,9]. In parallel, marine-derived peptides are receiving increasing attention as sustainable sources of bioactive ingredients with potential antioxidant, anti-inflammatory, anti-photoaging, and skin-protective properties [9,10].

Fish processing commonly generates large volumes of low-value by-products. However, circular-economy goals can be achieved by converting waste into value-added products, and marine collagen aligns with this approach because it valorizes discarded fish biomass into high-value ingredients, thereby reducing environmental burdens and supporting zero-waste strategies [11,12,13]. Fish skin, particularly from salmon, is an abundant source that can be valorized as a promising source of collagen and collagen peptides, offering a scalable and low-cost feedstock for cosmeceutical actives [14,15]. In addition, salmon skin is typically used for the extraction of acid-soluble type I collagen, in which the triple helix can be retained [16]. Moreover, this collagen source is capable of transforming into low-molecular-weight collagen peptides (LMWCPs) through enzymatic hydrolysis [17]. These peptides offer several advantageous characteristics, including high bioavailability, low immunogenicity, and eco-friendly provenance compared to terrestrial collagen peptides [18]. Additionally, salmon-skin peptides offer practical advantages over bovine/porcine collagens since marine sources pose lower zoonosis concerns and fewer religious constraints, aligning with the circular bioeconomy valorization of seafood by-products [19].

At the cellular level, collagen peptides can upregulate ECM programs in dermal fibroblasts. In normal human dermal fibroblasts, collagen peptides increased COL1A1, ELN, and VCAN transcript levels and augmented collagen deposition, supporting a pro-matrix, pro-repair phenotype [6]. This aligns with broader evidence that ECM assembly and remodeling in fibroblasts are orchestrated by TGF-β signaling through canonical Smad pathways and complementary non-Smad routes [20]. TGF-β is a well-established inducer of type I collagen and has also been shown to stimulate elastin gene expression in skin fibroblasts. Although such molecular pathways are now well studied, the demonstration of this gene panel after LMWCPs treatment has not been addressed in prior collagen-peptide studies. Therefore, this study aimed to evaluate the effects of salmon skin LMWCPs on the mRNA expression of key ECM-related genes, including COL1A1, COL3A1, ELN, VCAN, and TGF-β, in BJ fibroblasts. The study further explored the relationship between the two-step hydrolysis process, physicochemical characteristics of the resulting LMWCPs, and the observed transcriptional responses.

## 2. Results and Discussion

### 2.1. Degree of Hydrolysis (DH)

**Figure 1:**
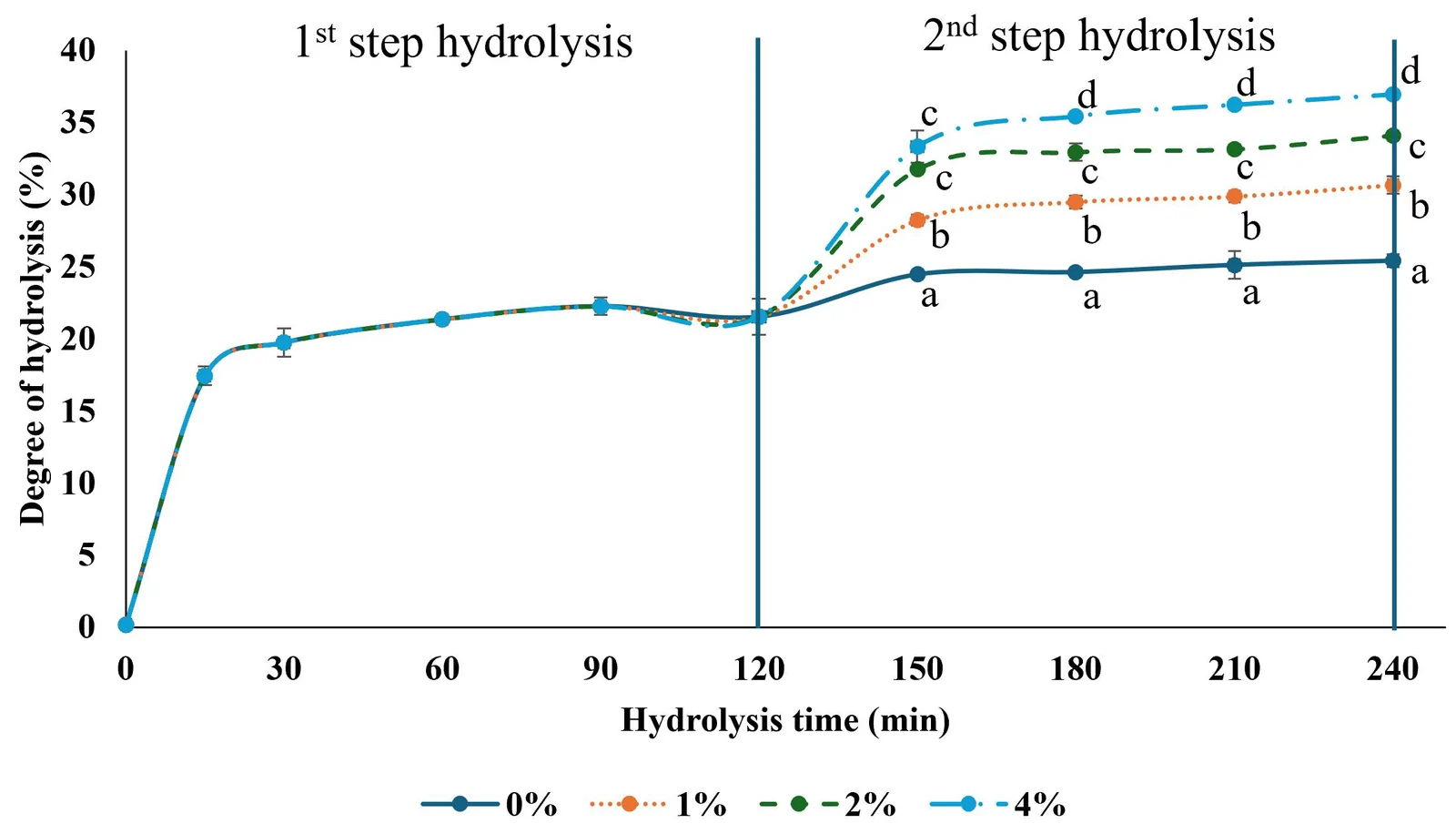
Degree of hydrolysis (DH) during two-step enzymatic hydrolysis of salmon skin collagen. The first step employed a combination of alcalase and papain as described by [22], followed by a second step using various concentrations of collagenase. Data are expressed as mean ± SD (*n* = 3). Different letters on the graph indicate significant differences (*p* < 0.05).

The degree of hydrolysis (DH) of salmon skin LMWCPs obtained in the first step of hydrolysis using the mixture of alcalase and papain up to 120 min increased as a function of time (Figure 1). The initial hydrolysis with the mixture of 4% alcalase and 3% papain at pH 8.0 resulted in a rapid increase in DH and DH reached around 22% within the first 30 min of hydrolysis. This initial phase likely reflects the efficient cleavage of peptide bonds in the amorphous, non-helical regions of collagen, which are more susceptible to proteolytic enzymes [21]. It was suggested that salmon skin matrix could be cleaved to a great extent when the mixture of enzymes alcalase and papain was used. Alcalase might more specifically hydrolyze collagenous matter in skin, while papain had broader hydrolytic activity toward other proteins retained in the skin matrix after pretreatment [22]. A plateau was detected during 60–120 min of hydrolysis, suggesting a temporary depletion of easily accessible cleavage sites.

After the first step of hydrolysis, collagenase at various concentrations was employed for further hydrolysis with different hydrolysis times (Figure 1). Collagenase at various concentrations (0–4% *w*/*w*) was added after 120 min of the first step of hydrolysis (time: 120 min). It was noted that a marked increase in DH was observed, especially when the collagenase concentration increased after 30 min of hydrolysis (time: 150 min). Higher DH was obtained when a higher level of collagenase was used. Collagenase at higher levels was able to cleave the peptides generated from the first step at high extent. This was because the first step of hydrolysis for 120 min could reduce the compactness of the collagen structure, which facilitated collagenase to enhance cleavage of peptides to a greater extent. Furthermore, longer hydrolysis time could favor the hydrolysis of peptides. The highest DH (36.93%) was observed for collagen peptide prepared with 4% collagenase at 240 min of the whole hydrolysis process. This result indicated that the initial one-step hydrolysis (alcalase/papain) produced a rapid DH rise but plateaued between 60 and 120 min, suggesting depletion of readily accessible sites. Introducing a second collagenase step overcame this plateau, driving DH to ~36–37%, which is associated with the generation of lower-molecular-weight peptide species [23]. These forms of the peptides are resistant to gastrointestinal digestion and are typically favored for dermal compatibility and intestinal absorption [24,25,26]. Moreover, the present work adopted a sequential two-step process combining alcalase/papain pre-hydrolysis followed by collagenase treatment. This approach was designed to enhance peptide cleavage within triple-helix regions without requiring chromatographic or membrane-based fractionation, thereby simplifying the process and reducing production cost while maintaining a low-molecular-weight profile suitable for further analyses.

In contrast, a single-step hydrolysate would be expected to contain a broader and, on average, higher-MW profile with fewer triple-helix-specific cleavages, potentially limiting bioavailability and formulation uniformity. Therefore, the LMWCPs prepared from salmon skin using a two-step process with a mixture of 4% alcalase and 3% papain for 120 min, followed by collagenase at a concentration of 4% for another 120 min, were selected and used for further production, analysis, and characterization. Although there was no marked difference in DH between 60 and 120 min of the first hydrolysis step, 120 min more likely enhanced the unfolding of peptides in the skin, thus favoring hydrolysis by collagenase use in the second step.

### 2.2. Characteristics of LMWCPs from Salmon Skin

**Figure 2:**
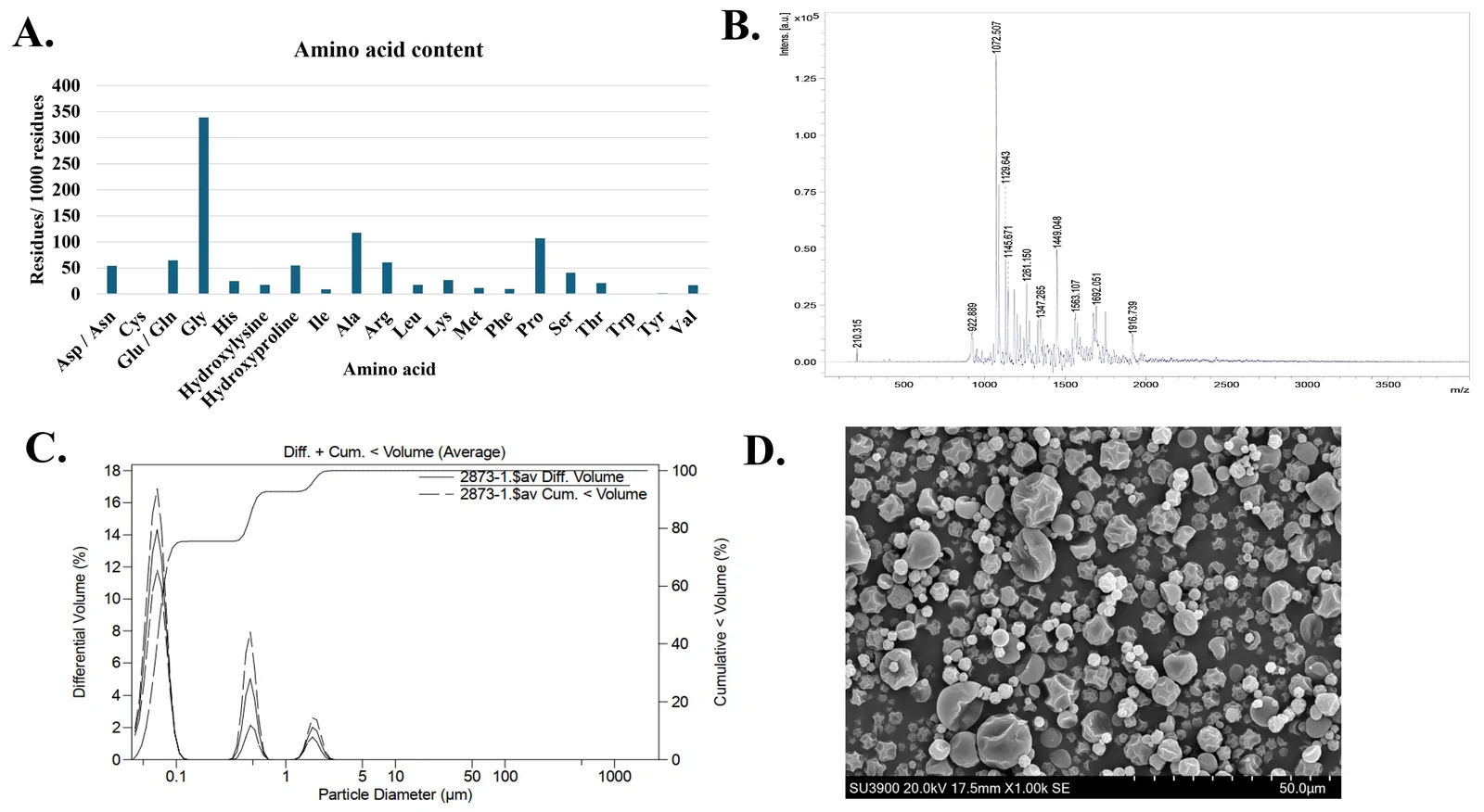
Characteristics of LMWCPs including amino acid composition (**A**), MALDI-TOF mass spectrum (**B**), particle-size distribution showing a mean diameter of 117 nm (**C**) and SEM images (**D**).

The spray-dried LMWCP powder contained 36.17% protein, 0.11% fat, 3.35% moisture, 1.02% ash, and 59.36% carbohydrate (Table 1). The powder had a protein/carbohydrate ratio of around 1:2. It was in accordance with the ratio of maltodextrin/peptide (2:1) before spray drying. Moreover, the amino acid composition revealed that the LMWCP powder consisted of glycine as the most abundant residue (339 residues/1000 residues). Amino acids, including proline (107 residue/1000 residues) and hydroxyproline (55 residue/1000 residues), were found in LMWCPs (Figure 2A). This result was related to the characteristics of mother collagen (Gly-X-Y repeats), in which glycine constituted approximately 1/3 of total amino acids. This characteristic was in line with other reports, which demonstrated the high content of glycine/proline/hydroxyproline in marine or salmon skin collagen. However, the literature indicates that porcine type I collagen typically exhibits a slightly higher Hyp content than fish sources (e.g., 13.3% in porcine and 10.4% in flatfish) [27]. This difference may be linked to the greater thermal/mechanical stability of mammalian fibers due to Hyp-stabilized triple helices.

MALDI-TOF results also revealed that peptides had MW mainly between 900 and 2000 Da, with a dominant peptide having MW at ~1072 Da (Figure 2B), consistent with hydrolyzed collagen rich in low-MW peptides (0.5–3 kDa). Chotphruethipong et al. (2021) documented that the hydrolyzed collagen from Asian seabass skin with low MW (<3000 Da) could promote osteoblast cell proliferation and enhance alkaline phosphatase activity during the first 7 days, while inducing mineralization at day 21 [28]. The composition and molecular-weight distribution confirmed the presence of predominantly low-molecular-weight peptide species in the LMWCP preparation. These characteristics provide physicochemical support for subsequent biological evaluation, although bioavailability and dermal efficacy were not directly assessed in the present study [29,30]. The presence of low MW peptides, particularly di-/tripeptides, is well documented to be found in human plasma after oral intake and to persist for hours, supporting bioavailability [31,32]. Such hydroxyproline-containing peptides have been linked to fibroblast migration and pro-matrix responses, providing a mechanistic rationale for downstream effects on ECM programs observed for fish-derived collagen peptides. The composition and molecular-weight distribution confirmed the presence of predominantly low-molecular-weight peptide species in the LMWCP preparation. These characteristics provide physicochemical support for subsequent biological evaluation.

For the mean particle size, the mean particle size was 0.117 µm (117 nm) with a zeta potential of −37.12 mV, corresponding to |ζ| = 37.12 mV (Figure 2C). This result suggested that the resulting powder was highly dispersible and electrostatically stabilized. It could be solubilized in water with ease due to the charge on the surface of peptides in LMWCPs. In general, an absolute zeta potential |ζ| ≥ 30 mV is considered a threshold for good colloidal stability [33,34]. This value is in line with the stable range commonly reported for cosmetic nano-systems, with absolute values around 30–40 mV, which are typically associated with good electrostatic stabilization and a low aggregation risk [33,35,36]. However, the value of zeta potential can shift after formulation due to pH, ionic strength, and excipients [35]. Taken together, the conditions selected by DH screening produced a narrow distribution of low-mass peptides that dispersed as sub-200 nm particles with a stable negative zeta potential, features that are expected to favor cellular compatibility and uptake [37,38,39]. However, these measurements characterize the dispersion behavior of the LMWCP preparation under the particle-size analysis conditions; solubility, sedimentation behavior, and cellular uptake under cell-culture conditions were not directly assessed.

Additionally, SEM images revealed that the powder was predominantly spherical, although some particles were dented or collapsed (Figure 2D). This structural appearance is typically observed in spray-dried products. For sugar-rich systems, in which rapid crust formation and the hygroscopic nature of the matrix, the powder generally had wrinkled microspheres. The literature shows that equant/spherical and smaller particles dissolve faster than needle/plate-like morphologies because they present greater accessible surface area and a thinner diffusion boundary layer, while anisotropic particles agglomerate and wet poorly [40]. Moreover, micronization/nanosizing enhances the aqueous solubility of diverse bioactives, demonstrating the generality of morphology–size effects [41]. Although the LMWCP preparation could be readily dispersed for cell treatment, its quantitative solubility and sedimentation behavior in complete culture medium were not determined and should be specifically evaluated in future studies.

### 2.3. Cytotoxicity of LMWCPs

**Figure 3:**
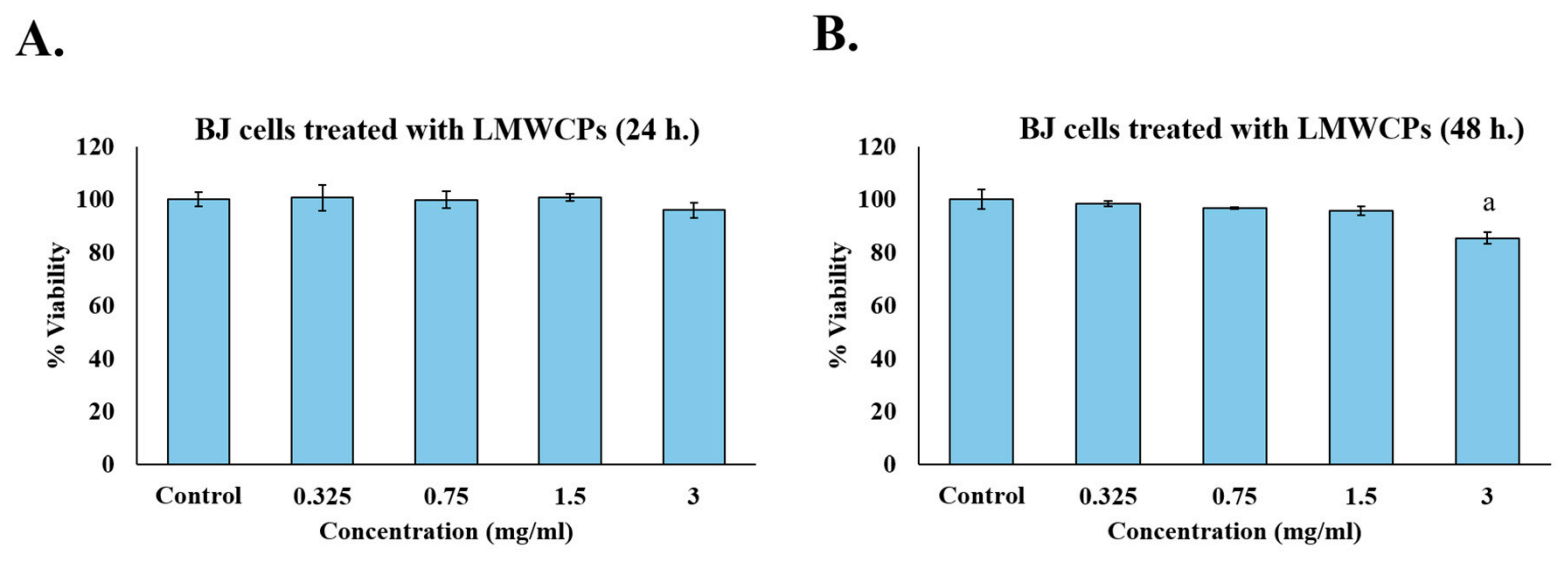
Cell viability of BJ fibroblasts treated with LMWCPs at different concentrations for 24 h (**A**) and 48 h (**B**), assessed by the MTT assay. Two independent cell-culture experiments were performed on separate days using a single hydrolysate lot, with three technical replicate wells per treatment condition in each experiment. Data are presented as mean ± SD. Vertical bars represent the SD. Letters on bars indicate significant differences (*p* < 0.05) according to one-way ANOVA followed by Tukey’s test.

In the cytotoxicity assay, BJ fibroblast viability remained comparable to untreated controls (≈97–102%) after 24 h of LMWCPs treatment at all tested concentrations of spray-dried salmon skin collagen peptides (Figure 3A), indicating no acute cytotoxicity. After 48 h, viability at 3.0 mg/mL declined to ~82–85%, whereas 0.325–1.5 mg/mL showed similar viability to that of the control (Figure 3B). These findings aligned with previous work showing that dermal cells were tolerant to marine collagen hydrolysates at sub-milligram to low milligram per milliliter ranges [6,42]. For example, Subhan et al. (2017) demonstrated that no cytotoxicity was observed when HaCaT keratinocytes were treated with up to 10 mg/mL of collagen extracted from fish scale for 24 h compared to the untreated control [42]. Moreover, a study by Martins et al. (2023) evaluated the cytotoxicity of collagen from Greenland halibut skin on L929 fibroblast cells with varying concentrations and showed that none of the tested concentrations of collagen (1, 2, 5, and 10 mg/mL) exerted toxic effects [43]. It is noted that the LMWCPs were non-cytotoxic across the tested range (MTT, ≤1.5 mg/mL over 48 h). The objective of this study was to define a safe, process-relevant dosing window, rather than to quantify an inhibitory effect. Consequently, an IC is not applicable to the endpoints. Together, these findings reinforce that LMWCPs are well tolerated across the tested concentrations.

Although several previous studies have consistently reported that collagen at relatively high concentrations (up to 10 mg/mL) does not induce cytotoxicity in fibroblasts or keratinocytes, this finding revealed a modest decrease in viability at 3.0 mg/mL after prolonged exposure. The small decrease in viability at 3.0 mg/mL after 48 h was likely due to the increased metabolic load of the cells from higher peptide concentrations over time, rather than true toxicity. This pattern is often seen in colorimetric viability assays when peptide levels are high [44,45]. Importantly, concentrations ≤1.5 mg/mL, where no cytotoxicity was observed, have been reported to show that human dermal fibroblasts treated with 0.01% (0.1 mg/mL) collagen peptides significantly upregulated the expression of COL1A1, ELN, and VCAN (all *p* < 0.05). The current results suggested that ≤1.5 mg/mL was an effective and safe concentration range for further ECM-related assays in BJ cells.

### 2.4. Gene Expression Analysis by RT-qPCR

**Figure 4:**
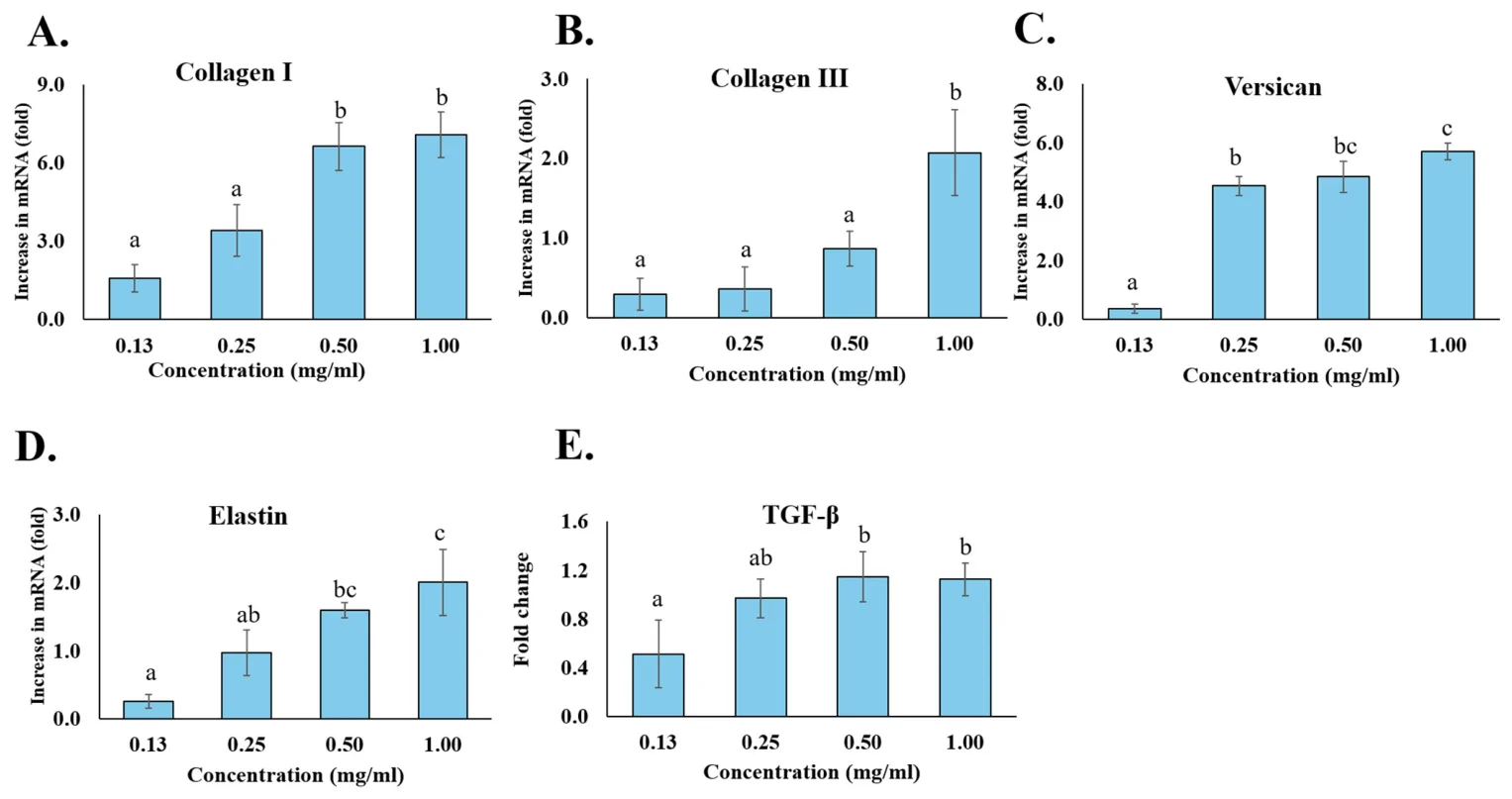
Relative mRNA expression levels of COL1A1 (**A**), COL3A1 (**B**), VCAN (**C**), ELN (**D**), and TGFB1 (**E**) in BJ human skin fibroblasts treated with LMWCPs at various concentrations. Two independent cell-culture experiments were performed using a single hydrolysate lot, with three technical replicates per treatment condition in each experiment. Vertical bars represent the SD. Different letters on bars indicate significant differences (*p* < 0.05) according to one-way ANOVA followed by Tukey’s test.

For RT-qPCR analysis, 1.0 mg/mL was selected as the highest concentration for subsequent RT-qPCR analysis to remain well within the cytocompatible range and to minimize potential confounding effects associated with reduced cell viability at higher concentrations. RT-qPCR analysis revealed that collagen peptides from salmon skin markedly stimulated the expression of ECM-related genes in BJ fibroblasts in a concentration-dependent manner. The most pronounced effect was observed for COL1A1, in which the LMWCPs at concentrations of 1.0 mg/mL and 0.5 mg/mL resulted in about 7.0-fold and 6.6-fold increases, respectively, compared with the untreated control (Figure 4A). Lower concentrations of 0.25 mg/mL and 0.125 mg/mL induced around 3.4-fold and 1.5-fold increases, respectively, demonstrating a dose–response relationship. In contrast, COL3A1 expression increased modestly (approximately 2-fold) only at the highest concentration (Figure 4B). VCAN expression also increased substantially by 5.7-, 4.8-, and 4.5-fold in the presence of LMWCPs at 1.0, 0.5, and 0.25 mg/mL, respectively (Figure 4C). ELN and TGF-β showed moderate increases (approximately 1.2–2.0-fold) across all tested concentrations, with relatively minimal variation at higher doses (Figure 4D,E).

These patterns indicated coordinated changes in ECM-related gene expression, with COL1A1 and VCAN showing the strongest transcriptional responses, accompanied by supportive induction of elastin and TGF-β. The strong dose-dependent upregulation of COL1A1 was consistent with TGF-β-mediated activation of fibroblasts, which promoted collagen synthesis via the Smad pathway [46,47,48,49]. A previous study showed that exogenous TGF-β enhanced the binding of transcription factors (CBF/NF-Y) to specific regulatory motifs in the COL1A1 promoter, indicating that TGF-β is associated with fibroblasts by driving COL1A1 expression and collagen synthesis [50]. Moreover, TGF-β is known to upregulate elastin gene expression [51,52]. The previous work demonstrated that TGF-β1 enhanced elastin production in cultured lung fibroblasts by increasing gene transcription and stabilizing tropoelastin mRNA [53]. Conversely, blocking endogenous TGF-β1 activity with neutralizing antibodies significantly reduced both tropoelastin protein and mRNA levels. This scenario supports the current finding and suggests that the observed ELN changes might be partly driven by the concurrent rise in TGF-β. Importantly, moderate TGF-β activation (approximately 1.2–2.0-fold) could enhance ECM production without tipping towards excessive fibrosis [54]. It was reported that sustained TGF-β activation is a central driver of fibroblast/myofibroblast activation and fibrosis across tissues, and is implicated in hypertrophic scars and keloids in skin [20,55].

Although the increase in ELN mRNA was modest, such transcriptional changes may warrant further investigation at the protein and matrix levels. Studies reported that even small increases in elastin synthesis are important for maintaining skin elasticity and resilience, particularly in aged fibroblasts where elastin turnover is slow [56,57]. In this study, ELN increased only modestly (approximately 1–2-fold), and the result suggested diminishing returns above 1.0–1.5 mg/mL, especially since 3.0 mg/mL produced a modest viability decline. However, elastic-fiber assembly is intrinsically slow. Tropoelastin must deposit on microfibrils and be crosslinked by lysyl oxidase (LOX), in which short 48 h assays can understate ELN outcomes [58]. To strengthen elastic-fiber endpoints without exceeding the safety window, future studies should extend exposure and add protein-level readouts (tropoelastin, desmosine/isodesmosine), and test pro-elastogenic co-factors, such as enhancing LOX activity or TGF-β cueing, which have been shown to promote elastin matrix formation [59,60]. The increased VCAN expression also suggested the enhanced synthesis of proteoglycans that regulate ECM organization, cell adhesion, and tissue hydration, further contributing to dermal structure development [7,61].

It should be noted that earlier studies mainly showed that collagen peptides can upregulate ECM markers in fibroblasts or mitigate UVB damage in vivo, but they rarely mapped a complete path from process to phenotype. In the present study, the two-step hydrolysis conditions were linked to the MALDI-defined low-molecular-weight profile, dispersion characteristics, and changes in ECM-related mRNA expression. BJ cells (ATCC® CRL-2522™; American Type Culture Collection, Manassas, VA, USA), derived from neonatal foreskin, normal human skin fibroblasts derived from neonatal foreskin, were selected as a well-characterized skin fibroblast model. BJ cells have previously been used to investigate ECM-related transcriptional responses to hydrolyzed collagen and peptide/amino-acid treatments [62]. This exploratory association provides a preliminary process-to-transcriptional-response framework for subsequent protein-level and functional validation. Moreover, this work is an early-phase, process-to-phenotype study, conducted with a single hydrolysate lot under process-relevant conditions. Future studies should include multiple, independently prepared batches and formal lot-to-lot comparability analyses to establish manufacturing reproducibility. The current efficacy evidence is also limited to mRNA readouts. Western blotting and in vivo experiments are needed to confirm biological relevance in a whole-tissue context.

Building on these in vitro findings, the next phase should advance through four linked steps. First, develop formulations and establish stability by incorporating LMWCPs into simple serum, gel, or cream bases, then monitor content, particle size, and zeta potential under both accelerated and real-time storage. Second, demonstrate manufacturing consistency by producing multiple independent batches with clear release specifications. Third, broaden safety assessment using reconstructed human epidermis/skin models to evaluate irritation and sensitization. Fourth, bridge mechanism to outcome by linking fibroblast gene responses to protein and tissue readouts, then confirm these effects in ex vivo human skin and small pilot studies. Together, these future steps provide a framework for further translational evaluation of salmon skin LMWCPs, contingent on confirmation of protein-level effects, biological function, safety, and manufacturing reproducibility.

## 3. Materials and Methods

### 3.1. Chemicals

Alcalase (EC 3.4.21.62) (2.4 unit/g) from *Bacillus licheniformis* and papain from papaya (*Carica papaya*) latex (EC 3.4.22.2) (3 unit/g) were obtained from Siam Victory Chemicals Co, Ltd. (Bangkok, Thailand). Collagenase (Cat no.: NATE-1594, food grade, activity > 100,000 unit/g) was procured from Creative Enzymes (Frankfurt am Main, Germany). Sodium hydroxide pellets (NaOH, CAS no.: 1310-73-2) and sodium chloride powders (NaCl, CAS no.: 7647-14-5) were purchased from KemAus™ (Phakanong, Bangkok, Thailand). Hydrochloric acid (HCl, CAS no.: 7647-01-0) was obtained from QReC (Auckland, New Zealand). Sodium dodecyl sulphate (SDS, CAS no.: 151-21-3) was obtained from Bio-Rad Laboratories (Hercules, CA, USA). All the chemicals used were of analytical grade.

### 3.2. Salmon Skin Preparation

**Figure 5:**
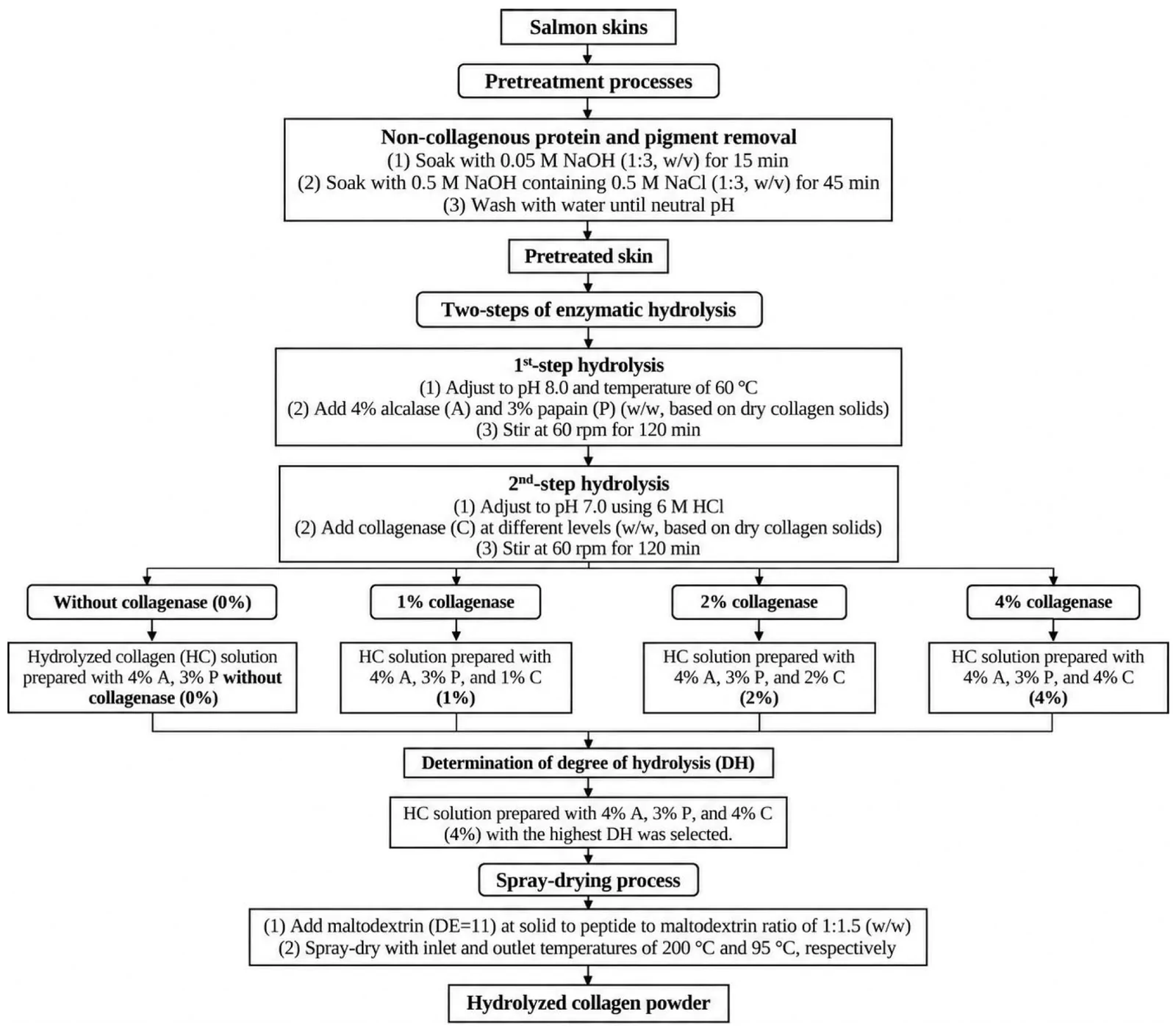
Schematic diagram for preparation method and treatment of hydrolyzed collagen from salmon skins.

Fresh salmon (*Salmo salar*) skins were manually descaled, scraped to remove residual flesh, and stored at −20 °C for 12 h. Frozen skins were cut into pieces with the aid of an electric sawing machine (Model W210E, Union Kitchen & Service, Bangkok, Thailand) and analyzed for moisture and total solids content according to the AOAC method, with an analytical method number of 950.46. For the removal of non-collagenous proteins and pigments, frozen skin was thawed in tap water at 26.0 ± 1.6 °C for 1 h before being weighed (20.01 ± 0.01 kg) and immersed in 0.05 M NaOH (60.03 ± 0.03 L) at a 1:3 *w*/*v* ratio with continuous stirring for 15 min. Skins were then collected, followed by the addition of 0.5 M NaOH containing 0.5 M NaCl (60.03 ± 0.03 L) at a 1:3 salmon skin-to-solution (*w*/*v*) ratio. The mixture was stirred gently at 150 rpm for 45 min to remove residual proteins and melanin. Thereafter, the solution was removed, and neutralization was performed using water (1:3, *w*/*v*), adjusted to pH 7.0 (7.00 ± 0.01) with 6 M HCl, and stirred for 10 min. The skins were rinsed with water 2–3 times until they were free of salts, and melanin-free skin was obtained. All the details are shown in Figure 5.

### 3.3. Preparation of Low-Molecular-Weight Collagen Peptides (LMWCPs)

Pre-treated salmon skins (10.00 ± 0.17 kg) were placed in a reaction vessel (capacity of 300 L, Euro Best Technology Co., LTD., Pathumthani, Thailand) and mixed with deionized water (21.97 ± 0.14 L) at a solid-to-water ratio of 1:2 (*w*/*v*). The mixture was heated to 60.00 ± 1.58 °C, and the pH was adjusted to 8.0 (8.00 ± 0.01) using 10 M NaOH. Enzymatic hydrolysis was performed in two steps to maximize hydrolysis as well as yield. The first hydrolysis was carried out following the previous work [22], which included digestion using alcalase (4% *w*/*w*, based on dry collagen solids) in combination with papain (3% *w*/*w*, based on dry collagen solids) for the broad-spectrum cleavage of peptide bonds. The mixture was stirred at 60 rpm for 120 min, and the pH was then adjusted to 7.0 (7.00 ± 0.01) with 6 M HCl. For the second digestion, collagenase (0–4% *w*/*w*) was then added, and the reaction was continued for an additional 120 min under constant stirring. To terminate enzymatic activity, the mixture was heated to 90 °C (92.5 ± 1.9 °C) and maintained for 15 min, then cooled to 29.0 ± 1.0 °C. During hydrolysis, the samples (1 mL) were taken and added to hot 5% SDS at a 1:1 (*v*/*v*) ratio and heated at 85 °C (85.5 ± 1.3 °C) for 30 min to terminate the reaction and solubilize total proteins and peptides. Degree of hydrolysis (DH) was measured as described by Benjakul and Morrissey [63]. A portion of each LMWCP was spray-dried, and the LMWCP powder samples were further analyzed. LMWCPs prepared with 4% alcalase, 3% papain, and 4% collagenase with the highest DH were selected for further spray-drying process. LMWCP solution was mixed with maltodextrin (2.66 ± 0.07 kg) at solid of peptide in LMWCP solution to maltodextrin ratio of 1:2 (*w*/*w*). The mixture was spray-dried using a spray dryer (SDE-5 Euro Workshop Euro Best Technology Co., LTD., Pathumthani, Thailand) with a feed rate of 1.03 ± 0.03 L/h. The inlet and outlet temperatures used were 200.3 ± 2.5 °C and 100.0 ± 1.6 °C, respectively. The LMWCP powder was collected, packed in an aluminum foil-laminated bag, and stored at −20 °C before analysis. All biological experiments in this study used a single hydrolysate lot from that process.

### 3.4. Chemical Composition Analysis

Moisture, protein, fat, and ash contents of LMWCP powder were analyzed using Association of Official Analytical Chemists (AOAC) methods (950.46, 920.153, 960.39, and 928.08, respectively) [64]. The conversion factor used for the calculation of protein content was 5.55, based on the Food and Agriculture Organization/World Health Organization (FAO/WHO) [65].

### 3.5. Amino Acid Analysis

Amino acid composition of LMWCP powders was determined in 4.0 M methanesulphonic acid under reduced pressure at 110 °C for 22 h to prevent the oxidation of tryptophan. The hydrolysate was neutralized with 3.5 M NaOH, diluted with 0.2 M citrate buffer (pH 2.2), and analyzed using an amino acid analyzer (JLC-500/V AminoTacTM, JEOL USA Inc., Peabody, MA, USA).

### 3.6. Particle Size Analysis

The mean particle size of different LMWCP powder samples was analyzed using a liquid particle size analyzer (LPSA) (Model LS 230, Beckman Coulter^®^, Fullerton, CA, USA), following the procedure described by Idowu et al. (2019) [66]. Each sample was initially dispersed in ethanol and introduced into the instrument under continuous stirring. Measurements were taken five times consecutively. The volume-weighted mean diameter (d43), which reflects the mean size of a sphere having the same volume, was calculated.

### 3.7. Scanning Electron Microscopic Analysis

A scanning electron microscope (SEM) (X-Max80; Oxford, UK) was used. The samples were coated with gold and examined in secondary electron mode at a 20 kV accelerating voltage. Images were captured and recorded at a magnification of 1000× using AZtec Software (version 2.4, Oxford Instruments plc, Abingdon, UK).

### 3.8. Size Distribution Analysis

The molecular weight of LMWCP powder was determined by MALDI-TOF following the method of Benjakul et al. (2018) [67]. In brief, the protein concentration of the sample was adjusted to 0.1 μg/μL with 0.1% trifluoroacetic acid (TFA) and mixed with an equal volume of alpha-cyano-4-hydroxycinnamic acid (CHCA) matrix before being applied onto a MALDI steel target plate (MTP 384 ground steel plate, Bruker Daltonik, GmbH, Bremen, Germany). The Autoflex Speed MALDI-TOF (Bruker, GmbH, Bremen, Germany) mass spectrometer equipped with a 337 nm nitrogen laser was used.

### 3.9. Cytotoxicity Assay in Normal Fibroblast Cells

Human fibroblast BJ cells (ATCC CRL-2522) were cultured in Eagle’s Minimum Essential Medium (EMEM; Gibco) supplemented with 10% (*v*/*v*) fetal bovine serum (FBS) and incubated at 37 °C in a humidified incubator with 5% CO_2_. Cytotoxicity of LMWCP samples toward BJ cells was evaluated using the 3-(4,5-dimethylthiazol-2-yl)-2,5-diphenyl tetrazolium bromide (MTT) assay with the Cell Proliferation Kit I (Roche Diagnostics, Basel, Switzerland), following the manufacturer’s instructions. BJ cells were seeded into 96-well plates at a density of 1 × 10^4^ cells/well and incubated for 24 h. LMWCP solutions at final concentrations of 3.00, 1.50, 0.75, 0.325, and 0 mg/mL were added to the wells, followed by incubation for an additional 24 h and 48 h. Subsequently, 10 µL of MTT reagent was added to each well, and the plates were incubated for 4 h before adding 100 µL of solubilization solution. Plates were further incubated for 16–18 h, and the absorbance was read at 570 nm using a FLUOstar^®^ Omega microplate reader (BMG Labtech, Offenburg, Germany). Two independent cell-culture experiments were performed using the same hydrolysate lot. Within each independent experiment, each treatment condition was assessed in three technical replicate wells.

### 3.10. Quantitative Real-Time PCR

To evaluate the effect of LMWCP samples on ECM-related gene expression, BJ cells were seeded into 6-well plates at a density of 2 × 10^5^ cells/well and cultured for 24 h. Cells were then treated with LMWCPs at concentrations of 1.00, 0.50, 0.25, and 0.125 mg/mL, while untreated cells served as the control. After 24 h of incubation, total RNA was extracted using the RNeasy Mini Kit (Qiagen, Hilden, Germany). RNA purity and concentration were determined using a NanoDrop spectrophotometer (Thermo Scientific, Waltham, MA, USA). One microgram of total RNA was reverse transcribed into cDNA using the QuantiNova Reverse Transcription Kit (Qiagen, Hilden, Germany). Quantitative real-time PCR (RT-qPCR) was performed on a QuantStudio 5 system (Thermo Fisher, Waltham, MA, USA) to measure expression of GAPDH (glyceraldehyde-3-phosphate dehydrogenase), COL1A1 (collagen type I alpha 1), COL3A1 (collagen type III alpha 1), ELN (elastin), TGF-β (transforming growth factor-beta), and VCAN (versican). The sequences of the primers are demonstrated in Table 2. Specificity of amplification was confirmed by melting curve analysis. Relative expression levels of target genes were calculated using the Pfaffl method, using GAPDH as the internal control. Two independent cell-culture experiments were performed using the same hydrolysate lot. Within each independent experiment, each treatment condition was analyzed in three technical replicates.

### 3.11. Statistical Analysis

Completely randomized design (CRD) was implemented for the entire study. All statistical calculations were conducted using SPSS version 26 (IBM, Armonk, NY, USA). Normality of residuals was assessed by Shapiro–Wilk with Q–Q plot inspection, and homoscedasticity by Brown–Forsythe tests. Analysis of variance (ANOVA) followed by Tukey’s test was used for comparison. Results with *p*-value < 0.05 were categorized as statistically significant. For both MTT and RT-qPCR assays, two independent cell-culture experiments were performed using a single hydrolysate lot. Each treatment condition was measured in three technical replicates within each independent experiment. Technical replicates were averaged within each independent experiment before statistical analysis.

## 4. Conclusions

This study establishes a process-to-phenotype framework for salmon skin LMWCPs and shows a broadened extracellular matrix gene signature with strong collagen and versican responses under non-cytotoxic conditions. The findings point to practical next steps in simple topical bases, with attention to stability of particle attributes and zeta potential, and to bridging from gene expression to protein outcomes that support product claims. Moreover, industrial scalability remains a key milestone, including multi-batch manufacture with clear release specifications, control of raw-material variability, and compatibility with routine drying and packaging workflows. In the present study, important limitations include use of a single hydrolysate lot and reliance on short in vitro exposures and gene-level endpoints. Future work should verify protein-level effects, extend to ex vivo human skin, and pilot clinical evaluations. Beyond reproducing known ECM stimulation, this study defines a preparation together with a broadened and mechanism-linked ECM signature. These findings provide a preliminary basis for future translational evaluation of salmon skin LMWCPs rather than evidence of translational or cosmeceutical readiness. This valorizing of fish processing by-products aligns with circular bioeconomy goals and the growing demand in cosmeceuticals for sustainable, non-mammalian, and traceable actives. Further validation of protein-level effects, biological function, safety, manufacturing reproducibility, and performance in more physiologically relevant models will be required before potential skin-health applications can be established.

## Figures and Tables

**Table 1:** Chemical composition of the spray-dried salmon skin LMWCP powder on a wet basis.

Parameters	Values * (%)
Protein content	36.17 ± 0.06
Fat content	0.11 ± 0.01
Moisture content	3.35 ± 0.01
Ash content	1.02 ± 0.00
Carbohydrate content	59.36 ± 0.06

* Values are means ± SD (*n* = 3).

**Table 2:** Primers used for gene expression analysis.

Primers	Sequences (5’-3’)		Sources
Collagen I	Forward	TGG CAA AGA AGG CGG CAA AG	[59]
	Reverse	AGC ACC AGC AGG ACC ATC AG	
Collagen III	Forward	GAT GGT GCT CCT GGT AAG AAT GG	
	Reverse	GGG TCC TGT GTC TCC TTT GTC A	
TGF-β1	Forward	ACT ACT ACG CCA AGG AGG TCA C	
	Reverse	AGA GCA ACA CGG GTT CAG GTA	
Elastin (ELN)	Forward	GGT GGC TTA GGA GTG TCT GC	[6]
	Reverse	CAC CTA CAC CTG GAG CCT TG	
Versican (VCAN)	Forward	CTG GTC TCC GCT GTA TCC TG	
	Reverse	ATC GCT GCA AAA TGA ACC CG	
GAPDH	Forward	GTG GTC TCC TCT GAC TTC AAC A	[68]
	Reverse	GCT GTA GCC AAA TTC GTT GTC A	

## Data Availability

The original contributions presented in the study are included in the article and further inquiries can be directed to the corresponding author.
